# A Feasibility Study of the SAFE Pilot Program: A University–School Board Partnership in Ontario

**DOI:** 10.1177/10497315231159059

**Published:** 2023-02-28

**Authors:** Jane E. Sanders, Ariel Seale, Victoria Lewis, M.K. Arundel, Rick Csiernik

**Affiliations:** 1 School of Social Work, King's University College at Western University Canada, London, Ontario, Canada

**Keywords:** university–community partnership, social work, information and communication technology, parental mental health, feasibility study

## Abstract

The Support and Aid to Families Electronically (SAFE) pilot program was developed through a community–university partnership to support parents of elementary students in Ontario, while providing stable practicums for social work students in the midst of COVID-19 restrictions. **Purpose:** The aim of the current study was to examine the feasibility of the SAFE pilot program as a mental health support to families by examining three feasibility objectives: demand, acceptably, and implementation. **Method:** Qualitative data from interviews, focus groups, and qualitative surveys involving service users, social work students, referring school board and university professionals (*n* = 37) were examined. **Results:** Demand for SAFE extended beyond the pandemic. A high-level of acceptance of SAFE was identified. Areas of success and considerations for implementation are outlined. **Discussion:** This study provides practice guidance on implementing this unique program, with potential to address gaps in service provision and the ongoing crisis in field education.

Research by Georgiades and colleagues in 2019 found that 18%–22% of children and adolescents in Ontario met criteria for a mental health disorder, with behavior and anxiety disorders the most common; only around a quarter of these children received mental health services, most often through their school. That same year, COVID-19 emerged, forcing widespread physical distancing and restrictions that profoundly impacted the delivery of mental health services and education from preschool to post-secondary. Nearly 1.6 billion learners representing over 90% of students around the globe were impacted by school closures (UNESCO, 2020). Schools in Ontario were among those most profoundly affected, closed to in-person learning from March 14, 2020, to May 15, 2021.

These closures, however necessary, placed a heavy burden on parents and caregivers who were suddenly and unexpectedly required to balance work, childcare, and education in the midst of increased financial stress, health concerns, and social isolation; all as access to support abruptly changed (Avena et al., 2021; Chiappini et al., 2020; Cowie & Myers, 2021; Evans et al., 2020; Gonzalez & MacMillan, 2020; Imran et al., 2020; Kandula & Wake, 2022; Kumar & Nayar, 2021; McNeil et al., 2023; Pollard et al., 2020; Yue et al., 2022). Moreover, COVID-19 exacerbated disproportionate preexisting social issues. Children with special education needs or those in families with fewer financial and social resources were disproportionately impacted by school closures and the burden to raise and educate children disproportionately fell on women (Gadermann et al., 2021; Lee, 2020; Mete Yesil et al., 2022; Radomski et al., 2022).

The shift to remote service provision also caused significant disruptions to social work education. Practicum placements provide an essential component of social work education through which students gain practice skills while being supervised by professional social workers in the field (Bogo, 2018; CSWE, 2015; Olson-Morrison et al., 2019). The additional resource drain when shifting to remote service provision resulted in the closure of a number of planned practicum placements, compounding pre-existing difficulties securing sufficient practicum opportunities (Grise-Owens et al., 2016; Morley & Clarke, 2020).

The Support and Aid to Families Electronically (SAFE) pilot intervention was developed through a community–university partnership between the King's School of Social Work and the Thames Valley District School Board (TVDSB). SAFE was designed to address parental stress and mental health through free and immediate online counselling, while providing stable remote practicum placements for social work students. The purpose of the current study was to examine the feasibility of the SAFE pilot program as a support to families (Sanders et al., 2022).

## Review of the Literature

COVID-19 disrupted the majority of field placements resulting in premature termination or transition to remote projects, limiting opportunities to engage in direct social work practice, previously a hallmark of practicum (Davis & Mirick, 2021; Drolet et al., 2021, 2022; McFadden et al., 2020). In response, many placement opportunities were supported and supervised within the university. Most of these university-led placement opportunities encompassed student-led learning opportunities, research projects (Lomas et al., 2022; Morley & Clarke, 2020), or simulations, in which students engage with actors in the role of client, as an alternative to field placements (Jefferies & Mason, 2021; Kourgiantakis et al., 2019; Kourgiantakis & Lee, 2020; Tortorelli et al., 2021).

Community partnerships filled a unique need where students remotely connected and provided psychosocial support to isolated populations during the COVID-19 pandemic (O’Keeffe et al., 2022). Despite this, we know of very few placement opportunities similar to SAFE. Two notable programs were developed during COVID through the Factor-Inwentash School of Social Work at The University of Toronto. One is the Talk It Out Counseling Clinic, a community partnership model through which MSW students provided short-term counseling and community workshops with an anti-racism and trauma-informed service orientation, targeting those who face multiple barriers to health and equity and those who belong to Black communities (Fang et al., in press). The other is #SafeHandsSafeHearts, a brief, peer-delivered eHealth intervention employing MSW interns and other peer counselors to deliver the online intervention (Newman et al., 2021; P. A. Newman, personal communication, January 25, 2023). A third program was developed at the University of South Florida School of Social Work, in which the Problem Management Plus intervention was taught to and delivered by MSW students at the preexisting BRIDGE clinic, a student-run free clinic (Galea et al., 2022). SAFE services a differing social and community need, focusing on parents in need of psychotherapeutic support.

Levels of parental stress were exacerbated during the COVID-19 Pandemic (Chung et al., 2020; Fontanesi et al., 2020; Javed et al., 2020; Prime et al., 2020; Romero et al., 2020; Spinelli et al., 2020; Wiemer & Clarkson, 2022). Moreover, the importance of parent and caregiver involvement within child and youth mental health has been consistently identified (Arat & Wong, 2016; Hawke et al., 2021; Shucksmith et al., 2010; Wang et al., 2019). Caregivers to children with mental illness experience unique strains and burdens, and are at increased risk for deteriorating health outcomes, reduced socialization with friends, impacts on job performance, and strained familial relationships (Mendenhall & Mount, 2011). Both psychotherapy and psychoeducational psychotherapy to caregivers enhance caregiver strengths while decreasing caregiver stress (Mendenhall & Mount, 2011). A scoping review of 151 studies aimed at understanding factors associated with effectiveness of interventions to improve child and youth mental health found that stepwise, multi-domain approaches that incorporate the child or youth's social network, specifically parents, was important for effectiveness, and improved the parents’ own mental health while improving the mental health of the child (Bjerre et al., 2021).

A systematic review of approaches to supporting students at risk of poor mental health outlined the benefits of parental involvement in school-based approaches. There were also significant barriers noted to full parental involvement (Shucksmith et al., 2010). Such barriers cause tension and stress in caregivers’ attempts to access services, advocate, and provide mental health care for their children (Boydell et al., 2006; Miller et al., 2017).

## The SAFE Program

The SAFE program emerged in partnership with the TVDSB, one of the largest school boards in Ontario, with 132 elementary and 29 secondary schools covering a diverse rural and urban area. Referrals to the program came through TVDSB social workers and counselors. SAFE was designed to address significant gaps in community-based parent and caregiver supports during the first phase of the COVID-19 restrictions and school closures. SAFE provided parents and caregivers of school-aged youth access to no-cost, low-barrier support provided by social work students. The SAFE pilot program was open to all elementary schools within the TVDSB, although families in which there were custody or access disputes or current justice involvement, were not eligible for service. The program was initially intended to run from January to June of 2021, however additional funding was secured to offer an extension through July and August to families who requested ongoing SAFE services through the summer.

The SAFE program ascribes to a wholistic approach designed to meet service users where they are at through a comprehensive social work assessment, formulation, and collaborative treatment plan. This allowed for service provision that addressed the issues that families were dealing with through the flexible application of evidence-informed approaches across treatment modalities (Drisko & Grady, 2015). SAFE students were supported through weekly 2 h group supervision with a contracted and experienced registered social worker, regular access to their supervisor between supervision sessions as needed, a bi-weekly 2 h seminar class with students from other placements and led by their faculty consultant, regular access to seminar instructor/faculty consultant as needed, regular access to practicum office as needed, regular meetings with the research team, and continual peer support facilitated through an online chat group. The partnership between King's and TVDSB was fostered by regular meetings between the TVDSB's manager of professional services and the coordinator of field education for The School of Social Work at King's. This continuous communication allowed for prompt adjustment of the program as needed. An important example was the explicit agreement that if caseloads hit an upper threshold, the social work Field Education Office would communicate directly with the TVDSB to stop the flow of referrals, ensuring that referring professionals could predict a prompt response and would be advised if they needed to support families in other ways.

## An Integrated Knowledge Translation Approach to a University School Partnership

The development of the SAFE intervention and the current research is in line with an integrated knowledge translation (IKT) approach. Knowledge users within King's Field Education Office and the TVDSB identified a problematic gap in both supports available to parents and social work student learning opportunities, and were in a position to act (Bowen & Graham, 2009). Consistent with IKTs goal of generating evidence-based, relevant, and usable knowledge, these knowledge users continued to be involved as members of the research and writing team along with the SAFE students (Preyde et al., 2013).

SAFE is a unique example of a university-community partnership. While such partnerships can present unique challenges, there are significant benefits to the university, community, agency, students, and service users resulting in shared resources, expertise, and addressing mutual needs (Dulmus & Cristalli, 2012; Forchuk & Csiernik, 2021; Lewis et al., 2016). The SAFE program, for example, was designed to: provide increased access to social work services through the development of this pilot intervention; take pressure off community agencies, in particular the school board (SB); allow students to provide up to date evidence-informed practice while honing their skills; and to create stable strong placements for social work students.

## The Current Study

The objective of the current study was to understand the feasibility of the SAFE pilot intervention related to four main stakeholder groups: families, King's social work students, King's Field Education Office, and for the referring SB (Thabane et al., 2010). Feasibility studies are indicated when there is little data on a specific intervention and when community partnerships need to be increased or sustained, both of which were the case with the SAFE program (Bowen et al., 2009). The development of SAFE as an intervention and educational opportunity is described elsewhere (Sanders et al., 2022).

The current study focused on the feasibility of the SAFE pilot intervention as a service to families, including the following feasibility objectives in line with Bowen et al. (2009). Firstly, we explored demand for SAFE, considering the extent to which this program is likely to be used beyond the pilot. Secondly, we sought to understand acceptability: how satisfying, appropriate and perceived positive or negative SAFE was thought to be by the four main stakeholder groups. Thirdly, implementation, considering the success or failure of implementation of the pilot, the resources needed for ongoing implementation, and the factors affecting ongoing implementation (Bowen et al. 2009). The qualitative interview data gathered from the four key stakeholder groups informed the first feasibility objective, demand, supported by descriptive data on how many families were served, who was served (family demographics) and presenting issues. Qualitative data also informed the second and third feasibility objectives, acceptability and implementation; the latter explored the success of, and considerations for, implementation.

## Method

The current study was a feasibility study using thematic analysis (TA) of qualitative data (Braun & Clarke, 2006, 2021). TA is a flexible approach for organizing, analyzing, and conceptualizing patterns or themes noted within the data set. The qualitative data from interviews and surveys were analyzed through the six stages of TA to inductively “organize, interrogate, and interpret” the data (Braun & Clarke, 2021, p. 4). Qualitative data was the primary data collected to ensure that this study reflected the realities of the community and the practice setting of SAFE and to provide meaningful opportunities for the key stakeholders to express their experiences of the SAFE intervention (Bowen et al., 2009).

### Study Participants and Other Data Sources

A total of 37 individuals participated in the study (*n* = 4 parents, *n* = 7 SAFE students, *n* = 4 King's professionals, *n* = 22 SB professionals) (see demographic Tables 1 to 4). It is notable that the sample was predominantly White and female. The qualitative data was supported by descriptive data on how many families were served, who was served (family demographics) and presenting issues (see Table 5).

**Table 1.** Parent Participants (*n* = 4).

Characteristics	Sample (%)
Gender identification	
Female	4 (100%)
Race	
White	4 (100%)
Age	
30–40	4 (100%)
Religion	
Not applicable	4 (100%)
Relation to TVDSB student	
Mother	4 (100%)
SES**^a^** Mean neighborhood household income (Canadian average 82,436.48 CAD)	
0–61,146.21 CAD	3 (75%)
61, 146.22 CAD–78, 076.58 CAD	0 (0%)
78,076.59 CAD–94,299.98 CAD	0 (0%)
94,299.99 CAD–116,561.33 CAD	1 (25%)
116,561.34 CAD–856,675.04 CAD	0 (0%)
Neighborhood educational attainment ^b^ (Canada average 31.52%)	
0%–19.23%	0 (0%)
19.24%–27.72%	3 (75%)
27.73%–37.11%	0 (0%)
31.12%–49.05%	1 (25%)
49.06%–100.00%	0 (0%)

^a^ Based on family postal code data.

^b^ Percentage of household population 25–64 years by educational attainment / household population 25–64 years/university certificate, diploma or degree at bachelor level or above.

**Table 2.** King's Participants (*n* = 4).

Characteristics	Sample (%)
Title/Position	
Field education administrative assistant	1 (25%)
Coordinator of field education	1 (25%)
Field supervisor	1 (25%)
Faculty consultant	1 (25%)
Years in role	
1–10	2 (50%)
11–20	1 (25%)
21 and above	1 (25%)
Education	
BA, MA	1 (25%)
BA, BSW, MSW	2 (50%)
BSW, MSW	1 (25%)
Gender identification	
Female	4 (100%)
Race	
White	4 (100%)
Age	
40–50	1 (25%)
51–60	1 (25%)
61–70	2 (50%)
Religion	
Christian (including non-practicing)	3 (75%)
Not applicable or not identified	1 (25%)

**Table 3.** TVDSB Staff Participants (*n* = 22).

Characteristics	Sample (%)
Profession	
School support counsellor	9 (40.91%)
Social worker	4 (18.18%)
Social worker/attendance counsellor	7 (31.82%)
Not identified	2 (9.09%)
Education (TVDSB staff have multiple diplomas)	
Social work	13 (59.09%)
Social work and psychology	1 (4.55%)
Social work, art therapy and psychology	1 (4.55%)
Psychology and art therapy	1 (4.55%)
Child and youth work	3 (13.64%)
Master of education in counselling	1 (4.55%)
Not identified	2 (9.09%)
Years practicing in role	
1–10	6 (27.27%)
11 and more	13 (59.09%)
Not identified	3 (13.64%)
Race	
White	18 (81.82%)
Prefer not to answer	1 (4.55%)
Not identified	3 (13.64%)
Gender	
Female	17 (77.27%)
Male	2 (9.09%)
Prefer not to answer	1 (4.55%)
Not identified	2 (9.09%)

**Table 4.** SAFE Student Participants (*n* = 7).

Characteristics	Sample (%)
Title/Position	
Student counsellor	6 (85.71%)
Student intake worker	1 (14.29%)
Years in role	
<1	7 (100%)
Education	
BSW student	1 (14.29%)
BSW, MSW student	1 (14.29%)
College degrees, Bed, MSW student	1 (14.29%)
College degree, BSW, MSW Student	2 (28.57%)
BA, MA, MSW student	1 (14.29%)
Not identified	1 (14.29%)
Gender	
Female	6 (85.71%)
Male	1 (14.29%)
Race	
White	5 (71.43%)
Prefer not to answer	1 (14.29%)
Not identified	1 (28.57%)
Age	
21–30	5 (71.43%)
31–40	1 (14.29%)
Not available	1 (14.29%)
Religion	
Agnostic	1 (14.29%)
Jewish	1 (14.29%)
Christian	1 (14.29%)
Prefer not to answer	1 (14.29%)
Not applicable or not identified	3 (42.86%)

**Table 5.** Demographics of Families Referred to SAFE (*n* = 59).

Characteristics	Sample (%)
Referred person's relationship to student	
Mother	44 (74.58%)
Father	1 (1.69%)
Mother and father	10 (16.95%)
Mother and stepfather	1 (1.69%)
Grandmother	1 (1.69%)
Not identified	2 (3.39%)
Gender identity of individual service users	
Woman	42 (61.76%)
Man	10 (14.71%)
Not identified	16 (23.53%)
Race of family	
Asian and White	1 (1.69%)
Indigenous and White	3 (5.08%)
Latinx	1 (1.69%)
White	37 (62.71%)
Not identified	17 (28.81%)
Religion of family	
Christian	9 (15.25%)
Muslim	3 (5.08%)
Not applicable	31 (52.54%)
Not identified	16 (27.12%)
SES **^a^**	41 (100%)
Mean neighborhood household income (Canadian average 82,436.48 CAD)	
0–61,146.21 CAD	17 (28.81%)
61, 146.22 CAD–78, 076.58 CAD	9 (15.25%)
78,076.59 CAD–94,299.98 CAD	8 (13.60%)
94,299.99 CAD–116,561.33 CAD	5 (8.47%)
116,561.34 CAD–856,675.04 CAD	2 (3.39%)
Not identified	18 (30.50%)
Neighborhood educational attainment **^b^** (Canada average 31.52%)	
0%–19.23%	22 (37.29%)
19.24%–27.72%	11 (18.64%)
27.73%–37.11%	1 (1.69%)
31.12%–49.05%	6 (10.17%)
49.06%–100.00%	1 (1.69%)
Not identified	18 (30.50%)

^a^ Based on family postal code data

^b^ Percentage of household population 25–64 years by educational attainment/household population 25–64 years/university certificate, diploma or degree at bachelor level or above.

#### Researcher Description

Our research team was made up of knowledge users in various roles in the School of Social Work including faculty consultant, professor, coordinator of field education, graduated students from SAFE and BSW and MSW research assistants.

### Participant Recruitment

The participants in this study were representative of the service users of SAFE and based on the same inclusion/exclusion criteria for involvement (Thabane et al., 2010). All students whose practicum was the pilot, all service users of the pilot intervention, all school board (SB) professionals eligible to make a referral, and all involved King's professionals were invited to participate between June 2021 and January 2022. Participants were contacted through email and parent participants were given $25 gift card as compensation for their time. Ethics approval was granted by the supporting university.

### Data Collection Procedures

Data were collected through semi-structured interviews and focus groups, each 60–90 min and conducted over Zoom by the lead author, with the exception of the student interviews which were conducted by a co-author. Given the ongoing challenges of COVID-19 for participants, two online surveys were delivered through Qualtrics. These surveys included open-ended text questions from the semi-structured interview and demographic questions, provided to those unable to attend an interview but interested in participating, one for SB professionals and one for parents. Consistent with the remote method of service provision, interviews were conducted over Zoom and audio-recorded with permission. Three parents, four professionals from King's, and seven social work students completed individual interviews via Zoom. Twenty SB professionals and one parent completed on-line surveys. Three focus groups were held: one consisted of two SB professionals, and two consisted of six of the SAFE students who had previously been interviewed. The individual SAFE student interviews were conducted at the completion of their practicum and the SAFE student focus groups were held three months later, after conclusion of the extended summer session of SAFE. Interviews and focus groups were transcribed and analyzed along with the qualitative content from the surveys, using TA. A pre- and post-clinical measure of parent stress was instituted in SAFE however, was not included in the current analysis as intended due to challenges in its consistent implementation resulting in available data for only one participant.

### Data Analysis

As per the first step of TA, three of the co-authors (J.S., A.S., V.L.) and one research assistant, familiarized themselves with the data. During step two, interview transcripts, focus group transcripts, and Qualtrics survey responses were line-by-line coded by two independent coders. The focus was on inductively emerging coding categories. In step three, codes and potential themes were identified for each subset of data. In the fourth step, themes were reviewed and inconsistencies were discussed. In the fifth step, the team collaboratively defined and refined the identified parent themes and co-occurring subthemes of the entire data set, with the feasibility objectives of the study in mind. As per step six, the analysis was finalized in the current manuscript. To reduce coder bias and enhance reliability, trustworthiness, and credibility (Anastas, 2004; Nowell et al., 2017), an audit trail was kept to document research decisions, and each interview, focus group, or survey was coded independently by two research assistants. In addition, the lead author conducted all interviews excluding the student participants and independently coded all interviews that she did not conduct, as well as over half of the surveys.

As part of the sixth step of the TA process, the lead author analyzed and organized the inductively derived themes to answer each of the feasibly objectives. To understand demand and acceptability, the related themes were identified and are detailed in the results section. Analysis of data for the first objective, demand, involved TA as described above, as well as descriptive analysis of demographic data: presenting issues, how many families were served, and family demographics. The third feasibility objective was examined by analyzing the qualitative data to explore the success of implementation and reviewing the resource demands of the program.

## Results

### First Feasibility Objective: Demand for SAFE

There was a total of 59 referrals to the SAFE pilot. Sixteen families did not follow through on their referral, seven families who completed the intake process did not engage in ongoing support, and 35 families received ongoing service with 11 of those continuing into the summer beyond the initial parameters of the practicum. Mothers were referred far more than any other caregiver group: Forty-two mothers (74.58%), 11 parental couples, one single father, and one grandmother were referred (see Table 5). A majority of the families referred to SAFE identified as White (62.71%). Forty-two percent lived in a neighborhood in the lowest of five income categories and 62% were in the lowest two, well below the Canadian average. Fifty-three percent resided in a neighborhood in the lowest of five categories of educational attainment.

Our TA of referral reasons found the most common referral reason involved child externalizing behaviors, aggression, and managing emotions (23 referrals); followed by anxieties (14); family stress related to maintaining daily routines, boundaries, screen time or sibling conflict (10); difficulties with online learning or school attendance (5); and finally, specific situations such as grief, gender transitioning, or neurodivergent supports (4); three referrals had no referral reason.

#### Low Referral Numbers Reflected Low Awareness of SAFE Pilot

SAFE students and King's professionals noted that for the initial pilot year of the program the referral numbers were lower than anticipated, however, they felt that this was a reflection of the limited awareness of the program within the SB, as one student noted, “only a few social workers who were doing referrals at the beginning, so it gave me the impression that not everyone knew it was an option” (ST07). King's participants noted the challenge of ramping up during the pilot stage of implementation, “it's always a slow start as you’re getting clients onboarded” (K01). Another noted, “the referral numbers were the concern. I was very surprised… are there still barriers that are preventing families from being able to access” (K02). The data in this study however, supported the idea that it will take time for the full complement of referring professionals to become familiar with SAFE. The following SB participant comment reflects what close to half of the SB professionals noted, “I didn’t have a lot of experience about what the program was and what steps they might take if there is a difficult client to engage and those sorts of things” (SB-FG01, R2). SB participants were clear that they “will keep referring as long as you keep offering this support, as the feedback I received from the family who invested their time with you were very satisfied” (SB26).

#### Complimentary Service Needed Beyond COVID-19

SAFE offered a complimentary service while filling a gap, a theme which informed both our demand and acceptability objectives. Supporting the work done in schools with children and increasing capacity to support the entire community was seen as relevant beyond the immediate response to COVID-19 that this pilot was initially addressing. As one school professional described, “our scope is so big of what we offer that the parenting is the one part that is really difficult. We might offer parenting programs, or we might offer drop-ins, but not the type of service that [SAFE is] offering” (SB-FG01, R1). Another SB participant noted the benefit of “somebody that's there supporting that parent through whatever the parenting needs are, that maybe aren’t necessarily related specifically into the school but do align” (SB-FG01, R2).

Moreover, participants noted the benefit for the entire system, as one King's professional observed, “the SAFE program is not only supporting the students, not only supporting the families, but it's providing resources for the social workers…trying to manage the students that are in the classrooms and the teachers and all the rest of it” (K02). The long-standing stress on services was seen as extending beyond the SB, “our community has wonderful clinicians and services, we just don’t have enough so there's huge wait lists” (K04).

Approximately half of the 20 SB professionals surveyed (yes = 9, maybe = 1) felt that SAFE should continue past COVID-19 stating, “there will be a definite need in the community” (SB26), “[SAFE] helps address huge waitlists for services” (SB23), and “we are going to need more going forward not less” (SB02). The SB focus group was particularly clear about the ongoing demand for SAFE, “the objective of the program is exactly what our families need, and I would say that the stress, if we relate it back to why the program was developed, I think we’ll still have the reason to have the program. I would like to see it continue” (SB-FG01, R1).

### Second Feasibility Objective: Acceptability of SAFE

Overall, there was high acceptance of SAFE services, as demonstrated in this parent statement, “[my social work student counsellor] was amazing, like just with the ideas…to help our son get through his emotions, was amazing for us” (P05). Parent participants felt supported through their challenges and with their family's concerns; they felt their feelings and experiences were validated, they identified learning “a lot” about themselves, as well as being able to discuss their relationships and their own role in family dynamics. The SAFE students described their work as effective and valuable, as one stated, “I would say that it had a great impact on the families that were involved” (ST-FG01, R1). Our TA identified five themes related to perceived acceptability of SAFE: support to parents, new family dynamics, client directed and flexible, evidence-informed, and complimentary service that filled a gap.

#### SAFE Supported Parents to be Able to Support Their Children

An important theme was the support SAFE provided parents, enabling them to support their children. As one King's professional noted, “It's all about the kids, except that…in order to be all about the kids, you have to be all about the people that are most important in their lives, which are the parents” (K02). Parents felt that the focus on them gave them, “something that I could do. It was a thing that I could control” (P06). Rather than services being provided only for their child, this parent felt that the parent focus “was probably the biggest thing that made it kind of move forward as quickly as it did” (P06). SB participants had consistent observations, as one noted, “when caregivers are stressed, children become stressed” (SB26).

The impact of the SAFE intervention however, went beyond families into schools, as described by one parent, “so far this school year, our son hasn’t been having too many issues with other peers like he normally would” (P05). The feedback from SB professionals was consistent, as revealed in the following:

I could tell that each week…they were working on some really good parenting skills…one family in particular, had set goals and was doing some really concrete parenting and I could tell because she was sharing that with me… everything that this person was doing with her was working. (SB-FG01, R1)

The ripple effect of change that began within families was articulated by one participant,

when you support the family wholistically, the change that is created for everyone, including the teachers in that classroom, including the other kids in that classroom, including the social worker who has to support that classroom, including the principal, including the parents, the ripple effect of families that are struggling is significant. The ripple effect of providing support to those families is equally impactful. (K02)

#### Creating New Family Dynamics

COVID-19 brought significant stress to families, which one SB participant described as, “increased emotional responses from students and families, ongoing and persistent activated stress responses leading to decreased coping and more reactive responses” (SB08). It was particularly notable, therefore, when parent participants identified significant changes in family tension and communication through their work with SAFE. These changes created new family dynamics that for one parent included “apologizing,” “talks,” and “a lot less arguing” (P01). This participant went on to describe increased “confidence” and “having more tools in your toolbox…to deal with situations” resulting in change throughout their family, “my son, he communicates with me more, he wants to hang out with me more. The rage is going down” (P01). SAFE students noticed changes in parents as well, “more present parents, that they are more calm parents…more strengths-based language with themselves, there's less tension in the families” (ST05).

#### Service User Directed and Flexible Approach

The service user directed and flexible approach at the foundation of SAFE's practice philosophy emerged as another important aspect related to the perceived acceptability of the intervention. As a student described, “we really ran the spectrum of anything that they perceived to be affecting their parenting, any length of time” (ST04). One parent described the efficacy of this approach,

the whole reason for the referral was my son, we kind of branched out…and really looked at some of the roots of his behavioural problems and how that might relate to his relationship with his dad and my relationship with his dad…I was able to talk through so many issues. In a lot of ways, my feelings and my experiences were really validated. (P06)

SAFE students applied an evidence-informed model that allowed the assessment and formulation to guide the intervention approach, which was found to include cognitive behavior therapy, narrative approaches, brief solution focused work, psychodynamic informed, among other approaches. As one participant from King's noted, “the service is flexible to the needs of the families. It's based on assessing the families as opposed to ‘we provide this service, so this is what you’re going to get’” (K02). For the referring professionals this was very beneficial as they knew when they referred a family they would receive the services they needed, as one noted, “it was just a one-stop sort of shop to say, here's what you’re looking for, here's what you need, and we can help you out with that” (SB-FG01, R2).

#### Evidence-Informed Approach, Skilled Students

Overall, the service was described as evidence-informed and structured to ensure students were educated, skilled, and supported to do the work. Parents found the SAFE students were “knowledgeable” and “experienced.” One parent particularly noted the evidence-informed approach was important for them, “there were suggestions made, based on research” (P01).

Moreover, parents identified that a transparent and collaborative approach was employed in SAFE, in which students were clear about their limitations and how they were going to address these to meet the needs of a particular family. This approach increased trust in the program as one parent described,

I found it helpful to know upfront what her limitations might be and what she was going to do about those… that she was taking that back and talking to people who were more experienced than she was. So, I think all of that that was built into the program was really well thought out. (P06)

Moreover, the evidence-informed and transparent service provision built trust amongst the referring SB, as was discussed in the focus group, “I know the King's program and I know that there are people that the students consult with, and they have supervision regularly” (SB-FG01, R2), and “there was some confidence that I could help our families [by referring to SAFE]” (SB-FG01, R1).

#### Complimentary Service Provision That Filled a Gap

COVID-19 exacerbated an already stretched support system, increasing stress and workloads throughout the system, participants noted, “there is also a noticeable increase in the amount of school staff that require support, many feeling emotionally and physically exhausted, uncertain of the future, and some feeling burnt out” (SB06). Professionals articulated that the stress in the system resulted in “increased referrals because staff are overwhelmed” (SB26).

The SAFE program was seen as filling an important gap in service provision. As one SB participant noted, “sometimes within the school system it's busy and we’re doing a lot of work with the students and sometimes we don’t have the ability to connect with the parents and do some of that core work that this program is able to offer” (SB-FG01, R2). The SAFE program provided complimentary services that involved the family in student support, as the SB professionals noted, “because then I knew I could focus on the student, and we know that their families were part of a system but we’re only one person and we’re stretched really thin, and we have very many roles in our school” (SB-FG01, R1). Moreover, as one King's participant noted, SB professionals “know that the service is going to be responsive…it's been co-created with the school board to meet the needs of the families that they care about, so that's huge” (K02).

### Third Feasibility Objective: Implementation of SAFE

Participants identified five main areas as being important implementation considerations: the referral and intake process, communication, service delivery, accessibility, and the resources needed for implementation. Findings related to implementation, including strengths and areas for development, have been summarized in Table 6.

**Table 6.** Implementation Recommendations and Considerations.

Implementation	Strengths and recommendations	Considerations for program development
Referral and intake process	• Open to all schools • Low-barrier referral process • Prompt response to referrals • Dedicated intake worker • Referrals from school with direct access to families in need • Referrals vetted by SB	• Mitigate barriers created by referral through school board professionals • Website and flyers to improve access to information about SAFE for parents and other professionals • Online referral form
Communication across systems	• Intake process quick, easy, and responsive • Independent from school board	• Increase communication between referring professional and SAFE students
Service delivery	• Individual and co-parent virtual sessions • Client centered, assessment driven, flexible, evidence-informed, transparent and collaborative • Strong supervision and support for SAFE students • Flexible hours • Flexible practice approach	• Offer face-to-face as needed • Walk-in or group approaches • Accessible consent forms and other online paperwork • Continue protocols related to safety and confidentiality with virtual service • Barriers regarding internet access • Continue to weigh the benefits of a clinical assessment tool • Client cancellations and dropout rate
Accessible service	• Timely response at intake and ongoing service • No waitlist • Free • Unlimited flexible sessions • Virtual • Added summer sessions • Translation	• Cost of internet and technology • Student availability (specific days and for duration of placement) • Balancing caseloads and number of students placed with maintaining no waitlist
Resources	• Supervisor/field instructor • Materials and laptop • Internet for supervisor and students • Secure SAFE email addresses • Supervisor liability insurance • Cell phone and plans for SAFE • Secure virtual platform for clinical notes • Clinical level secure video conferencing platform • IT support • Marketing to promote program within the referring school board • Development of program materials such as intake forms	• Additional resources required for development and ongoing implementation of SAFE absorbed the practicum office to be accounted for

#### Referral and Intake Process

The SAFE program, including the referral process, was intentionally designed to be low-barrier for both families and the referring professionals, as one SB participant noted, “the referral form was perfect because it was short, it was quick, we could submit it right then and we heard back right away” (SB-FG01, R1). The SAFE students felt the separate intake role was important as “the first point of contact” (SB01), which decreased anxiety as students could, “prepare for the case that [the students] were going to take on, to feel some anxiousness relieved…knowing what they are stepping into” (ST-FG01, R1). Moreover, receiving referrals from the school helped to identify the most appropriate fit, as school staff identified, “being able to assess, is this an appropriate referral or not to make sure it's what is best for the family and for the program too” (SB-FG01, R2). Alternately, some felt that requiring referrals come through SB professionals created access barriers. One parent noted it took a year to connect their child to the school social worker, “to even get…to see the social worker…That's a barrier in itself which would create a barrier for [SAFE]” (P01). While the SB professionals felt they were in the best position to refer, they agreed, “if maybe they’re not as keen to refer to social work they may not be able to access this program” (SB-FG01, R2).

Some participants suggested “a self-referral system” (ST02) could increase accessibility. One parent suggested that SAFE should be advertised within and outside of schools so families and professionals know how to access the resource, “draw attention to it, where it can benefit others” (P01). SB participants suggested: “a flyer” (SB-FG01, R1), “a website I could access and refer parents to look at” (SB27), “advertising” (SB30), or “a poster for families” (SB28).

#### Communication Between SAFE and the Referring SB

All participants identified that the formal channels of communication, specifically the intake process, worked well, however, opportunities to increase communication still existed. The SAFE students providing ongoing support noted, “we never got to talk to any of those referring social workers” (ST02). Likewise, the SB participants were interested in opportunities to collaborate, “my hope was that we connect both with the counsellor and the family together, we could talk about what their goals would be, we can integrate it into the school” (SB-FG01, R2). In particular, SB professionals being advised if a family had followed through on a referral and the possibility of a collaborative final meeting, “client led…and bring in the school…so that they can remember what they were working on” (SB-FG01, R2). It was noted however, that maintaining independence for SAFE would be important, as one participant noted,

One of the benefits of SAFE is that it is outside of the school board. If families have a difficult relationship with their school or with the school social worker or the board… this is an arm's length support that they might feel more comfortable accessing. (K02)

#### Service Delivery

While virtual delivery was mandated because of the COVID-19 pandemic, it allowed flexible service provision. Moreover, there was consensus that virtual counselling provided benefit without sacrificing the therapeutic relationship, “I felt really connected and engaged” (P06). The online service helped eliminate other barriers including transportation, schedules, and childcare concerns participants identified. For example, parents said that the flexibility to log in where and when they needed to allowed them to schedule sessions during their lunch from work without losing time traveling, which as participants noted, “allows more people to use it, especially families without transportation” (P08) and “it's great for people in a rural area” (K04).

Participants noted however, the importance of confidentiality and risk when working remotely. One consideration was the challenge of providing service when there is a “lack of privacy in the home” (SB27). Parents noted that while the flexibility that virtual provided was a benefit, finding a quiet space was a challenge, “it does take away… just that you’re not in that quiet room or whatever, away from everything” (P01). Moreover, remote work required particular attention was paid to potentially “unsafe home environments” (SB08) such as situations of intimate partner violence (IPV).

Furthermore, participants acknowledged the need “to pay attention to the families that [online] might not work for” (K02). One parent participant identified that while not necessarily a barrier to service, remote was not the preferred method of receiving services, “I’m not an online person” (P05) and the SB professionals noted, “many families on my caseload do not have internet or reliable internet” (SB23). This concern was also noted by the SAFE students, in particular, “with the [online] consent forms” (ST06). Therefore, participants suggested that in the future, face-to-face options for some families might augment the service provision and suggested “community based hubs or coming into the school to meet a family member if there are barriers to virtual” (SB08).

Additional options to expand service delivery were suggested, such as, “walk-in days or self-referrals days…psycho-ed webinar style groups or parenting groups” (ST02), and, as one SB participant described, “group parent support?…I think that can be helpful and just allow the ability to recognise that you’re not alone” (SB-FG01, R2).

#### Standardized Assessment Tool

A standardized clinical assessment tool was integrated into the program to be administered at the outset and again at the conclusion of service. Although some students noted that the data that this tool generated was “helpful and beneficial” (ST05) and flagged the “high stress” of families appropriately (ST04), others found it “difficult to navigate the standardized tool that we were using and getting that to clients” (ST-FG01, R3), particularly with families for whom English was not the first language.

#### Accessible Service

As has been discussed throughout this paper, accessibility was foremost in the design of SAFE, which came through across the data. The combination of SAFE being “accessible, free and with unlimited number of sessions” (SB24), with “little to no wait time for service” (SB26) was highlighted. The efforts to eradicate a waitlist meant that “families…have access to services, access to support at a time when mental health is at its peak” (K02). The responsiveness of SAFE was particularly important during first waves of COVID-19, as one SB participant noted that wait times in the community had increased dramatically and few services were providing ongoing support, “waitlists are horrendous at most places, longer term services are more difficult to access as many services are based in crisis” (SB06).

Participants therefore noted that in addition to the ongoing communication between the university and the SB regarding case flow, an ongoing implementation consideration was to balance the number of students with the anticipated referrals to ensure a ratio that will allow timely access to service, while at the same time, “you don’t want to have too many [students] because then you’re going to have very few clients” (ST03). Additionally, SB professionals noted the challenges related to parent/caregiver follow through on a referral, and with scheduled appointments, as one SB participant observed, “some families are overwhelmed and the thought of having another service provider calling them or having to make time, seems to be a challenge” (SB30).

Further related to implementation, participants stressed the importance of SAFE as a free service, as one King's participant commented on the benefit of “having access to care that is remote, that is consistent, that is free and accessible” (K03). Participants did however, recognize that service users still had to manage the cost of internet and a device to log onto sessions. For some families, the school was able to provide “Chromebooks to families” (ST01).

Unlimited and flexible sessions emerged as another implementation consideration that increased accessibility. Parents and students had flexibility to schedule appointments that fit the service users’ schedule, as one parent identified, “I was able to conveniently schedule it around where [my child] would most likely be napping” (P01), and as one student noted, “the stuff you’re working on is pretty complex, and not every client or case is the same, so it's hard to say eight sessions that's your hard cut-off” (ST07). As noted, additional sessions were offered to families over the summer. The flexibility and responsiveness of the program was highlighted by one King's participant who noted the following about the summer extension, “It's been able to pivot and respond to the needs of the families and the school board in a way that [in] other organizations it would be impossible to do” (K02).

Finally, the SAFE program was able to minimize language barriers through interpreters. Both required paperwork and sessions were translated as one King's professional explained, “so that our clients could better access the services and meet with the students” (K03).

#### Resource Demands

The resource recommendations and considerations of the SAFE program are outlined in Table 6. For the pilot program the resource costs totaled $12,676 CAD. It is important to note that resources directed to the development and ongoing implementation of the SAFE pilot were absorbed by the Field Education Office and were not factored into the tally of expenses but should be taken into consideration.

## Discussion and Applications to Practice

The SAFE pilot intervention was a unique response to the increasing need of social work services for families, made all the more pressing by COVID-19. In terms of demand for the program, there were 59 referrals, and 35 of the families who were referred received services. The majority of referred families identified as White and it will be important to understand why families of color were significantly less likely to be referred. There was little in the current data to inform this question, however the homogeneity of the sample, including the referring professionals, is noteworthy; future study is warranted. The demographic information however, demonstrated that SAFE was reaching women, and families residing in lower SES communities, both of whom were disproportionately impacted by COVID-19 and likely to need ongoing support well beyond the easing of restrictions.

When considering the demand for SAFE beyond COVID-19, it is important to note that the most common referral reasons included concerns related to aggression and emotion regulation, followed by anxiety. Although both of these top referral reasons were likely exacerbated by the overall stress of the pandemic, these are consistent with the most prevalent mental health concerns for children and youth pre-pandemic (Georgiades et al., 2019), further indication of the ongoing relevance of SAFE beyond the immediate crisis of COVID-19. This is consistent with existent literature, examined in a recent systematic review of telemedicine health interventions with caregivers caring for children with mental health concerns (Dörttepe & Duman, 2022). This systematic review highlighted that interventions such as parent training and therapeutic-based supports had positive impacts on caregivers of children with mental illnesses.

Moreover, school and community partnerships that bring mental health services delivered by community-based agencies have long been used to augment school supports (Fazel et al., 2014). Interestingly however, referral numbers were lower than had been anticipated given the profound and disproportionate need identified within the community (Im & George, 2022; McNeil et al., 2023). SAFE student participants observed that referrals were coming from a select number of SB professionals, confirmed by the survey results, which indicated that information about SAFE was slow to spread. Generally, the SB participants expressed confidence in the SAFE program and the School of Social Work. Moreover, the demand for support within the community was clearly articulated, as was the way this program alleviated the strain on resources for both the service providers and service users. The SAFE program not only supported families but referring to SAFE relieved stress on the system. SAFE addressed a gap, complimented services provided by the SB, and demonstrated the importance of the university–school partnership that led to the design of the program.

A number of themes emerged related to the high acceptability of SAFE; these themes are important to inform the SAFE program and broader social work practice. Firstly, providing support to parents and caregivers allowed these service users to address not only their parenting, but their own mental health needs and created space for them to be better parents. Secondly, working with the parents enabled the creation of new family dynamics that decreased tension and improved communication, a significant finding in the face of the profound stress experienced by families at the time of this pilot (Kourgiantakis et al., 2022). Thirdly, SAFE was service user directed and flexible. Each subset of participants noted the importance of the approach to evidence-informed practice that engaged collaborative case planning and allowed the client and service provider to determine the most appropriate approach to the work. The findings lend support for an evidence-informed approach through assessment, formulation, and collaborative goal setting to determine the practice approach applied (Drisko & Grady, 2015). Fourth, the approaches to practice were evidence-informed and grounded in practice theory; students were perceived to be educated on practice, transparent about limitations, and well supported in the provision of service.

In exploring the implementation of SAFE, a number of practice recommendations emerged. The approach was service user centered, flexible, assessment based, evidence-informed, transparent, collaborative and, perhaps most importantly, free, with limited wait times. The focus on accessibility addressed preidentified barriers for parents accessing mental health supports (Boydell et al., 2006; Miller et al., 2017; Shucksmith et al., 2010). The implementation of the program incorporated strong professional social work supervision and support for the students. The referral process was low barrier for the SB professionals, who found the process quick and responsive. Moreover, the students found having a dedicated intake worker beneficial. Therefore, the intake process was streamlined for SB professionals, who did not provide extensive referral information knowing it would be gathered at intake, ensuring the students providing ongoing support were well prepared at initial engagement without unnecessarily burdening the referring professional. Having referrals vetted by the SB was seen as an important component, as they could facilitate engagement and assess fit and readiness for the program. However, some participants proposed that this requirement created an additional barrier to access and suggested finding ways to mitigate this. A consistent finding was that increased access to information about SAFE would help referring professionals, community professionals, and potential service users advocate for referrals and make best use of the program. Suggestions included a website, flyers, posters, and an online referral form. The fact that SAFE was independent from the SB was generally viewed as a strength, however, finding ways to appropriately increase communication between the referring professionals and SAFE was seen as beneficial both to service users and SAFE students’ learning. Suggestions included communicating regarding referrals that did not proceed and joint meetings upon termination.

SAFE services were delivered through individual and co-parent virtual sessions. The findings of this study add to the growing knowledge base related to virtual service provision (Ashcroft et al., 2022; Im & George, 2022; Mishna, Sanders, Fantus, et al., 2021; Mishna, Sanders, Sewell, et al., 2021; Voth Schrag et al., 2023). Consistent with other research (Holmes & Foster, 2012; Zainudin et al., 2020), this study found that online service provision increased accessibility to services without sacrificing service delivery or the therapeutic relationship. This is particularly important given the research that identifies the importance of the therapeutic relationship for client outcomes (Cameron et al., 2018; Falkenström et al., 2014; Wilmots et al., 2020). As an augment however, participants suggested face-to-face sessions, when assessed to be beneficial to the service user, would strengthen the program, particularly for those whom virtual posed a barrier to service; this finding is consistent with those that indicate varying needs among service users related to ICT provided services (Mishna et al., 2022). Moreover, while one study of the impact of remote service provision on the therapeutic relationship prior to the pandemic predominantly cited it to be equal to face-to-face service provision (Mishna, Sanders, Fantus, et al., 2021), the impact of this global disruption of face-to-face relational work warrants extensive additional study (Pascoe, 2022). Suggestions to augment SAFE services included more user-friendly remote forms, adding walk-in services, and parent psycho-ed or treatment groups.

SAFE students were educated on service user safety and confidentiality in the context of remote service provision, identified as an important ongoing consideration in our study and in the existing literature (Mishna, Sanders, Sewell, et al., 2021). Social work students require education on crisis management and risk assessment in social work curricula and field placements, including, reconnecting or locating clients if technology fails during a crisis, routine check of who is with a client (gauges confidentiality and safety issues, and assesses potential supports), how and when to contact emergency services, and guidelines for documentation and privacy legislation (Mishna, Sanders, Sewell, et al., 2021).

One of the most important aspects of SAFE was its attention to accessibility. Participants identified a number of factors that removed barriers: timely response, no waitlist, free, unlimited sessions, virtual, attending to barriers posed by technology, extended summer sessions, and translation. While the virtual nature of service provision was seen as beneficial, providing flexible opportunity for support and reducing barriers to access, it was suggested that services could be offered both virtually and face-to-face in future, as determined by each family. This idea was echoed in the research, noting that remote, face-to-face, and hybrid placements will be necessary into the future (O’Keeffe et al., 2022).

One of the most notable considerations was managing the case flow to ensure no waitlist, which required predicting the number of referrals and placing the appropriate number of students, considering both responsiveness of the program and student learning needs, all while taking into consideration potential drop-off rate for referrals.

Additional considerations for social work program development are gleaned from SAFE being conceived and implemented through a university–schoolboard partnership that aligned with IKT. This facilitated a prompt response to service user and referring professionals needs and constant communication about implementation and service delivery. As participants pointed out, “the service is going to be responsive…it's been co-created with the school board to meet the needs of the families.” Notably, this included direct communication of the status of caseloads and referrals to ensure no waitlist and extending the program into the summer. Given the identified challenges related to caseloads and accessibility, this type of partnership, embedded in an IKT framework, is seen as particularly beneficial to the ongoing success of the program. Even if SAFE were to expand beyond the initial SB partnership of the pilot program, it is recommended that the Field Education Office continue this model of partnership moving forward, and that the resources needed to facilitate this are protected. Related to this is the discussion of resource needs for the program. Specifically, the development and ongoing delivery of SAFE was an additional demand placed on the Field Education Office.

While the current qualitative study is not generalizable, the findings are consistent with extant literature and provide direction for future research. Participant engagement was a challenge, likely due to the additional stress on both parents and professionals during the COVID-19 pandemic. The online survey was developed in response to these stressors and as a qualitative data tool is not evidence based. While the current study provides a strong understanding of demand, acceptability, and implementation for the SAFE pilot program, this study took place during a unique time and further evaluation will help us understand the ongoing utility and feasibly of this program. In particular, it will be important to study barriers to accessing SAFE for communities not well represented in this pilot study, specifically Black, Indigenous, and people of color, as well as those for whom remote service provision may be a barrier rather than a facilitator to accessing service. Given the rapid rise of remote forms of social work service provision, ongoing research in this area is needed (Mishna et al., 2020; Mishna, Sanders, Fantus, et al., 2021).

## Conclusion

The current study furthers the knowledge base of university-community partnerships in social work. It provides practice guidance for social work and similar programs looking to address the challenge of securing strong placement opportunities for students during COVID-19 and beyond. This has the potential to not only design programs that fill important gaps in service provision but address the mounting and ongoing crisis in field education (Grise-Owens et al., 2016; Morley & Clarke, 2020).
